# Own or Donated Human Milk: Its Role in Today’s Society

**DOI:** 10.3390/nu18020247

**Published:** 2026-01-13

**Authors:** Marta Suárez-Rodríguez, Gonzalo Solís-Sánchez

**Affiliations:** 1 Neonatology Service, Hospital Universitario Central de Asturias, 33011 Oviedo, Spain; solisgonzalo@uniovi.es; 2 Outcomes-Oriented Cooperative Research Networks in Health (RICORS-SAMID), 33011 Oviedo, Spain; 3 Health Research Institute of the Principality of Asturias (ISPA), 33011 Oviedo, Spain; 4 Medicine Department, Faculty of Medicine, University of Oviedo, 33007 Oviedo, Spain

In recent decades, the relevance of breast milk has increased due to the growing recognition of its unique properties, advances in milk banks, and research into its therapeutic uses. It has also become a topic of social, economic, and ethical importance.

In a globalized world with rapidly advancing medical technology and persistent health inequalities, human milk is simultaneously an irreplaceable biological resource and an indicator of equity.

Breast milk is the gold standard nutrition for newborns, meeting their nutritional, immunological and developmental needs. Its unique composition is rich in nutrients, immunomodulatory factors, enzymes, hormones, complex oligosaccharides, and microbiota, and cannot be replicated by artificial formulas. Furthermore, its dynamic composition varies according to the stage of lactation, maternal health, and the newborn’s needs, making it biologically designed to optimize infant growth and health [1,2].

Due to this exclusive composition, breastfeeding provides irreplaceable and well-established benefits. Human milk provides essential nutrients to the growing infant and is associated with protecting children from infections and certain diseases, improving cognitive development, promoting mother–child bonding, and significantly enhancing maternal health outcomes [3].

For all these reasons, the WHO established in 1990 that “Human milk is recommended for the first six months of life for all infants, including premature, twin, and sick infants, with very rare exceptions, and it should be continued for at least the first year and beyond if desired by both mother and child.” However, recognizing its benefits does not mean ignoring the challenges of ensuring access. During the first six months, breastfeeding rates are strongly influenced by work-related, socioeconomic, cultural, and health factors.

This Editorial seeks to reflect on the role of both donated breast milk (DBM) and mother’s own milk (MOM), in a context where social demands, scientific evidence, and structural limitations converge.

The GREEN MOTHER study (Contribution 1), conducted in the northern metropolitan area of Barcelona, is a multicenter study that evaluated how infant feeding evolves during the first month of life and what factors promote or hinder breastfeeding. A total of 411 women were surveyed, and the study showed that the exclusive breastfeeding rate decreased from 79% at hospital discharge to 64% at one month of age. Analysis of associated factors revealed that older maternal age, higher educational attainment, and longer duration of maternity leave were associated with higher rates of exclusive breastfeeding. However, exclusive breastfeeding rates remain below international recommendations, highlighting the need to strengthen health education, professional support, and social policies.

Similarly, the study conducted by Czerwinska et al. (Contribution 2) in Poland during the first six months postpartum concluded that the rate of exclusively breastfeeding declines progressively during the first months of life. The main reasons for this decline were breast problems, physical and mental exhaustion, and, above all, a lack of support from healthcare professionals. This study again highlights the need to strengthen breastfeeding training for healthcare staff and improve policies to support these mothers.

The work of Thi Tran et al. (Contribution 3), carried out in 2020 at the Women and Children’s Hospital of Da Nang in Vietnam, studied the factors that influence the prolonged use of DBM instead of the use of MOM and established strategies to increase the use of MOM, such as supporting breastfeeding from pregnancy through postpartum, in addition to daily assessment of the need for DBM. They concluded that, after the implementation of these measures, the use of DBM was relatively short, with an average duration of 4 days, which was much shorter than in other hospitals in the country. Therefore, providing greater breastfeeding support is essential to increase its use.

Rodrigues et al. (Contribution 4) analyzed whether the sex of the newborn and a multiple pregnancy modified the macronutrient composition of breast milk during the first 6 weeks postpartum. A single-center retrospective study was conducted, and significant differences were found in breast milk composition according to sex in each pregnancy group (twins vs. singleton pregnancies). Furthermore, the study determined that the sex of the infant and twin pregnancies can significantly alter the composition of preterm breast milk. This study demonstrates that breast milk should be fortified in a personalized way according to the specific needs of the newborn. 

Ávila-Alvarez et al. (Contribution 5) investigated whether the type of breast milk (DHM or MOM) used in the first days of life in premature infants increased the incidence of bronchopulmonary dysplasia. A retrospective study was conducted, including 159 neonates born under 32 weeks and/or weighing less than 1500 g at birth. Although there are differences in milk composition due to processes such as pasteurization, neonates receiving DHM did not have a higher risk of developing bronchopulmonary dysplasia than those receiving MOM. 

Finally, the LayDI cohort study, conducted by Martin-Ramos et al. (Contribution 6), followed 1946 children born between April 2017 and March 2018, aiming to establish a relationship between psychomotor development in the first two years of life and breastfeeding. While some results suggested that breastfeeding is associated with better psychomotor development at 24 months, the effects were moderate. As seen in previous studies, socioeconomic factors may play a more significant role than breast milk in determining developmental outcomes.

This Editorial highlights how, despite the clear benefits of breastfeeding in the first months of life, exclusively breastfeeding rates depend on multiple factors, particularly the support of healthcare services. Therefore, we conclude that DHM is the best alternative when MOM is not available.
